# Polish Validation of a 14-Item Version of the Religious and Spiritual Struggles Scale (RSS-14): Factorial Structure, Psychometric Properties, and Clinical Correlates

**DOI:** 10.1007/s10943-023-01816-5

**Published:** 2023-04-25

**Authors:** Adam Falewicz, Małgorzata Szcześniak, Radosław Rybarski, Marianna Chmiel, Joshua A. Wilt, Beata Zarzycka

**Affiliations:** 1 Institute of Psychology, University of Szczecin, Szczecin, Poland; 2 Institute of Psychology, John Paul II Catholic University of Lublin, Lublin, Poland; 3 Department of Psychological Sciences, Case Western Reserve University, Cleveland, USA

**Keywords:** Religious/spiritual struggles, Measurement, Validation, Health, Well-being

## Abstract

Religious and spiritual (R/S) struggles are defined as the occurrence of tension, conflict, or strain that focus on matters of ultimate significance perceived by people as sacred. The widespread prevalence of R/S struggles and the growing demand for research in this area created the need for a brief tool. Recently, the 14-item form of the Religious and Spiritual Struggles Scale was developed and validated (Exline et al. in Psychology of Religion and Spirituality, 2022a). Given the significance of the empirical research on R/S struggles, we implemented the project of structure verification, internal consistency confirmation, reliability, and nomological validation of the Polish variant of the RSS-14 through three separate studies. With respect to the internal structure of the RSS-14, the CFA from three studies confirmed the good fit of the six-factor model, very similar to the one obtained in the original version of the tool. Moreover, both the total score and the subscales had high reliability and acceptable stability over the three studies. With respect to the nomological analyses, R/S struggles were related negatively to life satisfaction, presence of meaning in life, self-esteem, social desirability, religious centrality, and positively with search for meaning, God’s disengagement, poorer health indicators, sleep problems, stress, and cognitive schemas (this category was the new element of our research). Polish 14-item version of the Religious and Spiritual Struggles Scale seems a valuable tool to assess religious strains.

## Introduction

Research at the intersection of the psychology of religion and clinical psychology has shown multiple links between religiosity/spirituality (R/S), mental health, and well-being (Park, 2013). R/S remain a significant source of support for people in difficulty (Captari et al., 2022). However, researchers have also taken an interest in the negative experience associated with religion and spirituality (Abu-Raiya et al., 2018). Among potentially problematic phenomena, religious and spiritual struggles, which are defined as the occurrence of tension, conflict, or strain, that focus on matters of ultimate significance perceived by people as sacred, have received considerable attention (Pargament & Exline, 2021). In fact, R/S struggles are present in virtually every religious and spiritual tradition (Abu-Raiya et al., 2018; Phillips et al., 2012; Tabik et al., 2020), even among those who tend not to affiliate, or identify themselves as spiritual, but not-religious. Recently, the 14-item form of the Religious and Spiritual Struggles Scale was developed and validated (Exline et al., 2022a). This article presents its Polish validation using three separate studies, in which the structure of the tool is confirmed and various variables support its nomological validity.

## R/S Struggles are Relevant to Health and Well-Being

R/S struggles are associated with various kinds of distress. Several studies have demonstrated that intensified religious struggles explain the current levels of mental and physical health (Bryant & Astin, 2008; Exline et al., 2022a, 2022b), showing associations with indicators of an individual’s poorer adjustment. People who experience strong religious struggles tend to manifest increased anxiety, depression (Yıldırım et al., 2022), obsessive-compulsive disorder symptoms (Moroń et al., 2022), and a higher risk of suicidal ideation (Upenieks, 2022a). A study by Exline et al. (2022b) has revealed a link between spiritual struggles resulting from the COVID-19 pandemic and the attribution of a demonic source to vaccines for the disease. Moreover, currently experienced religious struggles are predictors of increased psychological distress (Cowden et al., 2022) and negative psychological functioning.

## Development of the Abbreviated Form of the RSS

The 26-item version of the Religious and Spiritual Struggles Scale (Exline et al., 2014) was constructed as a development of Brief RCOPE (Pargament et al., 1998) and the Religious Comfort and Strain Scale (Exline et al., 2000). Both scales measured only certain aspect of struggles, and the aim of the authors of the original RSS was to create a tool that would comprehensively capture six domains of struggle: divine, demonic, moral, interpersonal, doubt, and ultimate meaning.

The widespread prevalence of R/S struggles and the growing demand for research in this area created the need for a brief tool. A shorter measure, yet one that includes all subscales of R/S struggles, was developed in 2022 (Exline et al., 2022a), meeting this demand. The goal of creating the RSS-14 was to be internally consistent while maintaining a broad spectrum of content and an identical structure to the original version. The RSS-14, similar to its original 26-item version, assesses: 1) divine struggles which refer to one’s inner conflicts around religious belief and the personal understanding of God; 2) demonic struggles which imply the experience of being harassed or attacked by the devil or evil spirits; 3) doubt struggles that allude to difficulties people experience about the truth of the religious content; 4) ultimate meaning struggles which describe inner conflicts and tensions around a person’s ultimate purpose in life; 5) moral struggles that refer to situations in which individuals perceive their behavior to be incongruent with their own values and beliefs; 6) interpersonal struggles around religion reflect its social nature, referring to shared values which lead to conflicts around religious themes, relations, and leadership (Pargament & Exline, 2021).

The selection of the items in the process of developing the RSS-14 was not dictated solely by the value of the factor loadings but was based on a thorough qualitative analysis of the content of the items (Exline et al., 2022a). The authors tested various sets composed of the selected items for maximum reliability. This method proved that different subscales required different numbers of items (2 or 3). The 6-factor structure of the RSS-14 has been confirmed, and there is growing evidence to support the reliability and criterion validity of each subscale (e.g., Wilt et al., 2022). An abbreviated version of the scale has already been used in a Polish sample by Moroń et al. (2022), but the authors omitted the ultimate meaning struggle scale due to their research objectives. However, the Polish version of the scale has not undergone extensive validation efforts, which is the purpose of this paper. Therefore, the first aim of the current study was to confirm the structure of the newest version of the brief measure of the RSS-14 and verify its internal consistency (Exline et al., 2022a).

## Nomological Validity of the RSS

The next goal was to confirm the preliminary nomological validity of the Polish variant of the RSS-14. Several variables were taken into account. The first criterion for selecting these constructs was their potential relationship to R/S struggles, justified by theoretical premises and empirical research. Some of them have already been verified (satisfaction, meaning in life, central dimensions of religiosity, general health, and perceived stress) and matched variables from the article presenting the development and preliminary validation of a 14-item form of the RSS (Exline et al., 2022a). The second criterion concerns the novelty of the selected variables in connection with R/S struggles (self-efficacy, self-esteem, perceived divine (dis)engagement, social desirability, and cognitive schemas).

Examining the strength of the relationship between them and R/S struggles could help deepen our understanding of the latter. More precisely, people who experience religious difficulties in the form of guilt/fear, adverse emotions toward God, religious individuals, groups, or communities (Yali et al., 2019) tend to evaluate their overall quality of life as less satisfying. However, some authors found null associations between life satisfaction and moral struggles (Exline et al., 2014), and demonic, moral, and doubt struggles (Wilt et al., 2022). Since no correlation was found in majority of studies with R/S struggles and the 6 dimensions and life satisfaction, we adopted the hypothesis of the lack of an association between all the dimensions of the RSS-14 and SWLS.

Next, the nature of religious beliefs affects individual outcomes and the self-concept (Schieman et al., 2017). Different studies show that people who report religious tensions and view religiosity as a source of unpleasant emotions are also likely to manifest lower levels of self-respect (Szcześniak et al., 2022a). More specifically, anger toward God (Wilt et al., 2016), religious doubts, little sense of a supportive relation with Divinity (Schieman et al., 2017), negative religious coping styles (Park et al., 2018) are associated with a decrease in self-esteem. Based on the empirical evidence, we assumed that R/S struggles would correlate negatively with self-esteem and self-efficacy.

Several studies have documented that R/S struggles predict worse mental health (Upenieks et al., 2022b), increase the risk of mortality (Pargament et al., 2001), and are associated with poorer general health (Upenieks, 2022c) or well-being (Gilbertson et al., 2022). Therefore, we hypothesized that R/S struggles would correlate positively with problematic health dimensions.

There is some evidence that R/S struggles are associated with meaning. According to Zarzycka et al. (2020), efforts to find meaning in adverse events positively correlate with demonic and moral strains, and negatively with ultimate meaning. Exline et al. (2014) showed that all dimensions of religious struggle, except its moral and demonic dimensions, were linked to lower levels of meaning in life. In turn, Wilt et al. (2022) clarified that the presence of meaning correlated negatively with ultimate meaning struggles and positively with demonic struggles. Based on the results achieved so far, we hypothesized that R/S strains would correlate negatively with meaning in life.

Several studies have shown that struggles related to religious or spiritual experience occur in the ampler context of general religiosity (Zarzycka et al., 2020). It has been demonstrated that personal religiosity is a critical source of comfort (Zarzycka et al., 2019) and psychological adjustment (Power & McKinney, 2013). People with enhanced centrality of religiosity can solve their struggles and show greater improvement in mental health than those who have a lower level of centrality (Friedrich-Killinger, 2020). Thus, we assumed that R/S struggles would be inversely linked to the centrality of religiosity and God’s engagement, and positively associated with God’s disengagement.

Research has confirmed that R/S struggles often take place in conditions of stressful events (Zarzycka et al., 2018). People experience some problematic forms of religiousness which are considered a source of strain (Abu-Raiya et al., 2018) and inner conflict (Exline & Bright, 2011). It has been confirmed that people feel stressed when they think they have been forsaken by God and experience religious guilt (Zarzycka & Zietek, 2019). Considering the previous findings, we hypothesized that R/S struggles would positively correlate with subjectively perceived stress.

The relationship between early maladaptive schemas (EMS) and religiosity is evidenced by research on scrupulosity (Soltanmohammadlou et al., 2022). Religiosity has also been shown to moderate the relationship between EMS and dysphoria (Racine & Cecero, 2005). Religious coping styles (measured with RCOPE) have been found to be a moderator of the relationship between EMS and measures of psychological adjustment (D’Andrea, 2003). Although there is a lack of previous research on the relationship between R/S struggles and maladaptive schemas, we hypothesized that they would correlate positively since maladaptive schemas reflect some degree of disfunction (Jovev et al., 2004) and contribute to several psychological problems.

Religious self-report tools are susceptible to socially desirable responding (Jones & Elliott, 2017). In some studies, it has been confirmed that people tend to present themselves or their religion in an attractive manner (Abu-Raiya et al., 2018). From one side, some individuals over-report their religiosity (Jones & Elliott, 2017) since being a religious or spiritual person is perceived in a positive way. In contrast, when people experience religious struggles, they may try to under-report their strains to protect and present their religion in a favorable light (Abu-Raiya et al., 2018). In our study, we expected negative correlations between R/S struggles and social desirability.

## Overview of the Current Project

Given the significance of the empirical research on R/S struggles, we implemented the project of structure verification, internal consistency confirmation, and nomological validation of the Polish variant of the RSS-14 through three separate studies. Conducting a confirmatory analysis (CFA) on data from the three samples was aimed at verifying the structure of the tool in the Polish context and ensuring that the selection of items was not accidental. The snowball sampling was applied as a means of data collection. All the respondents gave informed and written consent prior to the study and were assured of the confidence of the information they provided.

## Study 1

### Participants

The research was conducted with the participation of 250 adults, whose mean age was *M* = 37.16 (SD = 10.71, range 18–71). The respondents were also asked questions about their personal experience with religiosity across a number of topics on a scale of 1–10 (Table 1). Only 16 participants (6.4%) reported that had not experienced any specific R/S struggle.

**Table 1:** Demographics categories (studies 1–3)

	Study 1	Study 2	Study 3
Demographic category	*(N* = *250)*	*(N* = *261)*	*(N* = *300)*
Sex			
Woman	212 (85%)	186 (71%)	204 (68%)
Man	38 (15%)	76 (29%)	96 (32%)
Religious affiliation			
Catholic	177 (71%)	212 (81%)	195 (65%)
Atheist	34 (14%)	16 (6%)	61 (20%)
Agnostic	27 (11%)	16 (6%)	26 (9%)
Other	13 (4%)	17 (7%)	18 (6%)
Residence			
Urban area above 100,000 inhabitants	177 (71%)	144 (55%)	144 (55%)
Urban area below 100,000 inhabitants	43 (17%)	64 (25%)	71 (24%)
Rural area	30 (12%)	53 (20%)	64 (21%)
To what extent do you:	M / SD	M / SD	M / SD
Believe God exists	6.80 / 3.56	7.60 / 2.95	5.89 / 3.39
Practice your faith	5.46 / 3.54	5.84 / 3.05	4.62 / 3.20
Believe in the existence of supernatural evil (e.g., devil, demon)	6.04 / 3.77	6.68 / 3.10	5.23 / 3.22
Believe that the following persons/entities are responsible for the religious/spiritual struggle you are experiencing			
God	4.89 / 3.69	6.12 / 3.25	4.69 / 3.34
You	7.94 / 2.46	7.75 / 2.18	7.72 / 2.32
Another person, a group of people	4.70 / 2.86	5.06 / 2.57	5.03 / 2.54
Devil, evil powers	4.18 / 3.33	4.71 / 3.12	3.67 / 2.83
Fate, luck, chance	4.42 / 2.88	5.19 / 2.68	5.27 / 2.71

### Measures

The RSS-14 is an abbreviated version of the Religious and Spiritual Struggles Scale (Exline et al., 2022a) and assesses six types of r/s strains: divine, demonic, interpersonal, moral, ultimate meaning, and doubt. Its reliability and all other measures used in Study 1 are presented in Table 2.

**Table 2:** Descriptive statistics (study 1—*N* = 250)

	M	SD	Skewness	Kurtosis	α
Div	4.31	2.37	2.20	4.48	.84
Dem	2.81	1.63	2.27	4.77	.89
Int	6.79	2.96	0.58	− 0.32	.63
Mor	4.78	2.25	0.41	− 0.88	.72
Dbt	3.65	2.16	1.22	0.46	.84
Ult	3.79	2.28	1.18	0.38	.85
RSS-14	26.14	8.96	0.93	0.49	.85
Sat	21.68	6.06	− 0.36	− 0.07	.86
Ses	30.57	6.31	− 0.58	− 0.07	.90
Som	14.30	3.61	0.85	0.20	.80
Anx	14.75	4.31	0.74	0.10	.86
Sdy	14.73	2.92	1.03	2.29	.84
Sde	9.79	3.67	1.67	2.38	.86
GHQ-28	53.59	11.73	1.06	0.83	.92

*Div* Divine, *Dem* Demonic, *Int* Interpersonal, *Mor* Moral, *Dbt* Doubt, *Ult* Ultimate meaning, *RSS-14* Religious/spiritual struggle overall, *Sat* Life satisfaction, *Ses* Self-esteem, *Som* Somatic symptoms, *Anx* Anxiety and insomnia, *Sdy* Social dysfunction, *Sde* Severe depression, *GHQ-28* General health overall

The Satisfaction with Life Scale (SWLS) is a self-report tool used to measure satisfaction with the respondent’s life as a whole. The scale adapted into Polish by Juczyński (2001) consists of five items. The respondents assess the accuracy of each statement in relation to their life by using a seven-point Likert scale (from 1—*strongly disagree* to 7—*strongly agree*).

The Rosenberg Self-Esteem Scale (RSES) adapted into Polish by Łaguna et al. (2007) is a self-report measure that assesses individuals’ appraisal of the quality of their lives based on their own distinct standards. The 10-item scale consists of five positively and five negatively listed statements. The participants indicate their answers on a scale from 1—*strongly agree* to 4—*strongly disagree*.

The General Health Questionnaire (GHQ-28) adapted into Polish by Makowska and Merecz (2001) is a tool widely used for assessing the mental health of adults. It contains 28 questions divided into four subscales: somatic symptoms, anxiety and insomnia, social dysfunction, and severe depression. The participants rate their health on a 4-point Likert scale where 0—*not at all*, 1—*no more than usual*, 2—*rather more than usual*, 3—*much more than usual*.

## Study 2

### Participants

The research was carried out with the involvement of 261 adults, whose mean age was *M* = 29.43 (SD = 11.51, range 18–71). As in the other samples, the participants were invited to determine their level of personal experience with religiosity on a scale of 1–10 (Table 1). Only 6 participants (2.3%) reported that had not had any experience of R/S struggle.

### Measures

We used the RSS-14 in its Polish version. The alpha values for the RSS-14 and other scales used in Study 2 are presented in Table 3.

**Table 3:** Descriptive statistics (Study 2—*N* = 261)

	M	SD	Skewness	Kurtosis	α
Div	5.97	3.18	1.02	0.10	0.86
Dem	3.41	2.03	1.38	0.84	0.88
Int	7.67	3.32	0.47	− 0.62	0.70
Mor	5.72	2.23	− 0.07	− 0.84	0.62
Dbt	4.70	2.37	0.64	− 0.64	0.84
Ult	4.86	2.56	0.43	− 0.98	0.90
RSS-14	32.34	10.97	0.49	− 0.23	0.87
Mpr	24.34	6.22	− 0.49	− 0.20	0.87
Mse	25.78	5.62	− 1.21	2.14	0.84
Pss	20.97	6.30	0.06	− 0.57	0.85
Dep	19.03	6.58	− 0.06	− 0.56	0.70
Anx	15.51	7.81	0.08	− 1.11	0.88
Sde	4.44	2.03	0.04	− 0.72	0.58
Gen	11.07	4.39	− 0.24	− 0.97	0.90
Gdi	10.11	4.27	0.35	− 0.51	0.85
Inl	8.40	3.19	0.17	− 0.86	0.90
Ide	11.52	3.83	− 0.97	− 0.28	0.94
Prp	9.98	3.63	− 0.57	− 0.82	0.86
Exp	7.83	3.34	0.05	− 1.04	0.92
Pup	9.21	4.21	− 0.09	− 1.38	0.90
CRS-15	48.92	17.40	− 0.40	− 0.99	0.96

*Div* Divine, *Dem* Demonic, *Int* Interpersonal, *Mor* Moral, *Dbt* Doubt, *Ult* Ultimate meaning, *RSS-14* Religious/spiritual struggle overall, *Mpr* Presence of Meaning, *Mse* Search for Meaning, *Pss* Stress, *Dep* Depression, *Anx* Anxiety, *Sde* Social Desirability, *Gen* God’s Engagement, *Gdi* God’s Disengagement, *Inl* Intellect, *Ide* Ideology, *Prp* Private Practice, *Exp* Religious Experience, *Pup* Public Practice, *CRS-15* Religious Centrality overall

The Meaning in Life Questionnaire (MLQ) adapted into Polish by Kossakowska et al. (2013) is 10-item self-report tool created to assess presence of meaning, related to feeling that one’s life is meaningful, and search for meaning, which refers to seeking meaning. Items are estimated on a 7-point Likert scale ranging from 1 – *absolutely untrue* to 7 – *absolutely true*.

The Perceived Stress Scale (PSS-10), in the Polish adaptation by Juczyński and Ogińska-Bulik (2009), contains 10 items that measure the assessment of situations subjectively considered stressful. Each item is scored on a 5-point Likert scale, ranging from 0—*never* to 4—*very often*.

The Social Desirability Scale (SDS), inspired by the Marlowe–Crowne Social Desirability Scale and developed by Wilczyńska and Drwal (1995), measures people’s tendency to present themselves overly positively or unfavorably, and their desire to be accepted by others. For the purpose of this study, a shorter version was used. It included the items with the highest discriminating power.

The Brief Measure of Perceived Engagement and Disengagement in Response to Prayer (PDED), adapted into Polish by Szcześniak et al. (2021), is a short 8-item measure which assesses people’s levels of confidence in God’s attention to their prayers. The PDED has two dimensions. Divine engagement refers to a sense that God is listening and speaking to people. Divine disengagement connotes a lack of concern from God. Respondents rate the statements from 1—*never* to 5—*always*.

The Brief Screen for Depression (BSD) is a short measure designed for the initial assessment of depressive symptom of non-clinical patients. It was designed by Hakstian and McLean (1989) and consists of four items. The measure assumes a threshold at 21 points that may suggest a clinical level of experienced difficulties. In existing studies, the method received acceptable reliability levels. The current study used the method as translated by Zarzycka et al. (2022).

The Direct Behavior Rating-Scale Items Scale (DBR-SIS), as used in this study, was created by von der Embse et al. (2015). The scale is a brief measure to assess the level of anxiety. It consists of three items reflecting social, cognitive, and physiological aspects of anxiety. Respondents choose options from 1 to 10, with 1 for *no anxiety* and 10 for *very high anxiety.*

The Centrality of Religiosity Scale (CRS-15) is a measure that assesses the personal importance and salience of religious constructs within personality. The original version of the scale was created by Huber and Huber (2012) and adapted into Polish by Zarzycka et al. (2020). The measure consists of 15 items grouped into five subscales—Intellect, Ideology, Private, Religious Experience, and Public Practice. Respondents answer on a 5-point Likert scale, where 1 means *not at all/never* and 5 means *to a great extent/very often*.

## Study 3

### Participants

The research was performed on a group of 300 adults whose mean age was *M* = 26.39 (SD = 10.54, range 18–64). As in the previous surveys, the respondents were asked to provide demographic information and their level of experience regarding God and religiosity (Table 1). Only 14 participants (6.6%) reported that had not had any experience of R/S struggle.

### Measures

The RSS-14 was used in Study 3. The internal consistency of the RSS-14 and other tools used in Study 3 are presented in Table 4.

**Table 4:** Descriptive statistics (Study 3–*N* = 300)

	M	SD	Skewness	Kurtosis	α
Div	5.15	2.71	1.23	0.63	0.83
Dem	3.22	1.75	1.49	1.71	0.81
Int	7.13	3.38	0.49	− 0.81	0.77
Mor	5.08	2.27	0.12	− 1.10	0.78
Dbt	5.03	2.78	0.46	− 1.10	0.93
Ult	4.31	2.46	0.86	− 0.36	0.87
RSS-14	29.93	10.73	0.50	− 0.46	0.88
Sef	27.25	6.03	− 0.48	0.27	0.91
Emd	10.11	5.63	1.34	1.40	0.86
Aba	14.47	6.92	0.60	− 0.62	0.87
Mis	13.25	6.44	0.79	− 0.15	0.87
Soi	12.74	6.52	0.87	0.02	0.89
Def	10.24	6.00	1.38	1.35	0.90
Fai	12.07	6.53	0.95	0.09	0.91
Inc	11.06	5.13	0.98	0.51	0.78
Vul	12.18	5.98	0.87	− 0.05	0.82
Enm	9.77	4.57	0.96	0.48	0.78
Sub	11.61	5.45	0.87	0.18	0.79
Ssa	14.80	5.56	0.42	− 0.33	0.78
Emi	12.50	5.95	0.76	− 0.18	0.81
Unr	15.39	6.43	0.38	− 0.71	0.83
Ent	13.29	4.87	0.62	0.10	0.68
Ins	14.29	5.87	0.38	− 0.62	0.81
Adm	15.42	6.46	0.36	− 0.74	0.84
Pes	14.28	6.66	0.55	− 0.61	0.86
Spu	12.22	5.71	0.88	0.39	0.85
YSQ	229.75	82.66	0.59	− 0.25	0.98

*Div* Divine, *Dem* Demonic, *Int* Interpersonal, *Mor* Moral, *Dbt* Doubt, *Ult* Ultimate meaning, *RSS-14* Religious/spiritual struggle overall, *Sef* Self-efficacy, *Emd* Emotional deprivation, *Aba* Abandonment, *Mis* Mistrust/abuse, *Soi* Social isolation, *Def* Defectiveness/shame, *Fai* Failure, *Inc* Incompetence/dependence, *Vul* Vulnerability to harm, *Enm* Enmeshment, *Sub* Subjugation, *Ssa* Self-sacrifice, *Emi* Emotional inhibition, *Unr* Unrelenting standards, *Ent* Entitlement, *Ins* Insufficient self-control, *Adm* Admiration seeking, *Pes* Pessimism, *Spu* Self-punitiveness, *YSQ*  Overall score

The General Self-Efficacy Scale (GSES), adapted into Polish by Juczyński (2001), is a self-report instrument used to measure a sense of perceived self-efficacy and optimistic self-belief. The one-dimensional scale consists of 10 items. The respondents assess each of the ten statements by using multiple-choice answers on a 4-point scale that ranges from 1—*not at all true* to 4—*exactly true*.

The Young Schema Questionnaire (YSQ-S3-PL) is a measure of cognitive schemas (*Early Maladaptive Schema—*EMS; Young et al., 2006). The version utilized in the current research is a 90-item abbreviated version of the original, 232-item tool (Young & Brown, 2005), adapted into Polish by Oettingen et al. (2018). The measure is designed to assess the 18 EMSs: emotional deprivation, abandonment, mistrust, social isolation, defectiveness/shame, failure, incompetence, vulnerability to harm, enmeshment, subjugation, self-sacrifice, emotional inhibition, unrelenting standards, entitlement, insufficient self-control, admiration seeking, pessimism, and self-punitiveness. The items are rated on a scale from 1 (*completely untrue of me*) to 6 (*describes me perfectly*).

## Procedure and Data Analysis

The IBM SPSS Statistics 20.0 software was applied to calculate the descriptive, reliability (Cronbach’s alpha), and correlational statistics in all 3 Samples. The AMOS 21.0 software with Maximum Likelihood Estimation was used to confirm the structural model of the RSS-14 based on several goodness of fit indices. The assessment of reliability and validation of method was conducted using the approach recommended by Koenig and Al Zaben (2021).

First, all the variables were checked for the normality of distribution. We assumed the values of skewness and kurtosis between ± 2 as signs of a normal distribution. Since we expected intercorrelations between the dimensions of the RSS-14, the variance inflation factor (VIF) and a tolerance value were calculated to assess the degree of collinearity in a multiple linear regression model. A cutoff of 5.0 for the VIF and a tolerance value to 0.1 were adopted as indicators of a potential problem of collinearity (Field, 2017). The Mahalanobis distance (*χ*^2^ test with respective degrees of freedom and *p* < 0.001) and Cook’s distance (close to 1) were examined to detect the occurrence of potential multivariate outliers.

The six-factor solution was specified, using CFA. To obtain a more holistic view of the goodness of fit, the following global model indices were measured: the *χ*^2^ test not significant; Minimum Discrepancy per Degree of Freedom (χ^2^/df ≤ 3); GFI ≥ 0.9; AGFI ≥ 0.9; NFI ≥ 0.9; CFI ≥ 0.9; TLI ≥ 0.9; RMSEA ≤ 0.08; LO RMSEA ≤ 0.05; HI RMSEA ≤ 0.08; and SRMR ≤ 0.08. The threshold for the value factor loadings for the items was 0.50 and higher (Chou et al., 2017).

## Descriptive Statistics—Studies 1–3

Table 2 shows descriptive statistics obtained in Sample 1 for all the subscales of the RSS-14 (with the overall score), life satisfaction, self-esteem, and all the dimensions of the GHQ-28 (with the overall score). The values of skewness and kurtosis ranged from − 0.88 to 4.77, exceeding the values of ± 2 seen as indicators of a normal distribution. Therefore, Spearman’s rank-order correlation was used (Table 8).

Table 3 presents the descriptive statistics calculated in Sample 2 for all the subscales of the RSS-14 (with the overall score), presence of meaning, search for meaning, stress, depression, anxiety, social desirability, God’s engagement, God’s disengagement, overall score of religious centrality, and its dimensions (intellect, ideology, private practice, religious experience, public practice). The values of skewness and kurtosis ranged from − 1.38 to 2.14. Since only one value surpassed ± 2, we assumed that all variables had an approximately normal distribution. Consequently, Pearson’s correlation was used (Table 9).

Table 4 presents the descriptive statistics for all the subscales of the RSS-14 (with the overall score), self-efficacy, and all the dimensions of the YSQ-S3. All skewness and kurtosis values were within the range ranged from − 1.10 to 1.40 without exceeding the value ± 2. Based on these results, we assumed that the data are normally distributed, and we used Pearson’s correlation (Table 10).

## Multicollinearity and Outliers

The VIF values were below 5 in all three samples (ranging from 1.34 to 2.56 in Sample 1; from 1.13 to 4.61 in Sample 2; from 1.35 to 4.68 in Sample 3, except for the dimension of subjugation—5.27), and the tolerance statistics were higher than 0.1 (ranging from 0.389 to 0.744 in Sample 1; from 0.217 to 0.885 in Sample 2; ranging from 0.190 to 0.738 in Sample 3), suggesting that predictors were moderately associated with one another and supporting that multicollinearity was not a problem in Samples 1–3. The Mahalanobis distance method showed the presence of three cases of outliers in the data set (Sample 1), four cases (Sample 2), and 10 cases (Sample 3), reaching a significance level below *p* < 0.001. However, given that recomputation of the statistics with and without the extreme scores in all three samples yielded very similar results, we decided not to delete the observations with large residuals. The Cook’s distance values ranged between 0.000 and 0.116 (Sample 1), 0.000 and 0.076 (Sample 2), 0.000 and 0.261 (Sample 3) further confirming that outliers were not influential in the data.

## CFA Statistics

Table 5 displays the standardized loadings for the six-factor CFA obtained in Samples 1–3. A comparison of the Polish version of the questionnaire with the CFA results of the original RSS-14 (marked in parentheses) showed considerable similarity between the values of the three Polish samples and the original scale. All items loaded > 0.50 on their respective factors.

**Table 5:** Standardized loadings for six-factor CFA (Study 1–*N* = 250; Study 2–*N* = 261; Study 3–*N* = 300)

	Div	Dem	Int	Mor	Dbt	Ult
I2: felt as though God had abandoned me	.79/.84/.79 (.91)					
I3: felt as though God was punishing me	.91/.88/.77 (.90)					
I4: felt angry at God	.73/.76/.80 (.86)					
I7: Worried that the problems I was facing were the work of the devil or evil spirits		.98/.89/.86 (.95)				
I8: felt attacked by the devil or by evil spirits		.83/.88/.79 (.89)				
I10: felt hurt, mistreated, or offended by religious/spiritual people			.73/.80/.84 (.83)			
I13: had conflicts with other people about religious/spiritual matters			.54/.60/.68 (.76)			
I14: felt angry at organized religion			.58/.60/.68 (.69)			
I15: wrestled with attempts to follow my moral principles				.67/.55/.64 (.87)		
I18: felt guilty for not living up to my moral standards				.85/.82/.90 (.83)		
I24: felt troubled by doubts or questions about religion or spirituality					.88/.96/.94 (.90)	
I25: felt confused about my religious/spiritual beliefs					.84/.86/.92 (.85)	
I19: questioned whether life really matters						.81/.82/.93 (.92)
I20: felt as though my life had no deeper meaning						.93/.88/.83 (.87)

*Div* Divine, *Dem* Demonic, *Int* Interpersonal, *Mor* Moral, *Dbt* Doubt, *Ult* Ultimate meaning

Although the *χ*^2^ had statistical significance in all three samples (Table 6), thus suggesting rejection of the model, it is highly sensitive to the sample size (Watkins, 2021) since all three studies can be considered as large (over 200). Moreover, the Adjusted Goodness of Fit was slightly below 0.9 in samples 1–2. However, the other fit indices showed that the six-factor RSS-14 fits data well. Based on the results obtained, we assumed that the hypothesized model is correctly specified and consistent with the original version of RSS-14.

**Table 6:** Fit statistics for the six-factor CFA (Study 1–*N* = 250; Study 2–*N* = 261; Study 3–*N* = 300)

	*χ*^2^	*χ*^2^/d*f* ≤ 3	GFI ≥ 0.9	AGFI ≥ 0.9	NFI ≥ 0.9	CFI ≥ 0.9	TLI ≥ 0.9	RMSEA ≤ 08	LRMSEA ≤ 0.05	HRMSEA ≤ 0.08	SRMR ≤ 0.08
Study 1	148.592/62; *p* < .05	2.397	.923	.869	.911	.945	.919	.075	.060	.090	.056
Study 2	116.722/62; *p* < .05	1.883	.938	.895	.937	.969	.955	.058	.042	.074	.052
Study 3	133.489/62; *p* < .05	2.153	.941	.900	.941	.967	.952	.062	.048	.077	.044

## Intercorrelations Between the RSS-14 and Its Subscales

The correlations between the six factors resembled the results obtained in the original RSS-14 study (Table 7), except for demonic and ultimate meaning. Both factors were not associated with each other in Sample 1 and showed weak correlation in Sample 2. It is worth noting that in the study by Exline et al. (2022a), this correlation also showed the lowest, albeit significant value. In Sample 3, the tendency was observed between demonic and interpersonal strains.

**Table 7:** Intercorrelations between the overall RSS-14 and its subscales (Study 1–*N* = 250; Study 2–*N* = 261; Study 3–*N* = 300)

	RSS-14	Div	Dem	Int	Mor	Dbt	Ult
RSS-14	1						
Div	.65/.77/75***	1					
Dem	.36/.49/.54***	.43/.31/.41***	1				
Int	.68/.72/.66***	.27/.41/.36***	.02/.14*/.11^t^	1			
Mor	.66/.66/.69***	.39/.39/.42***	.37/.38/.47***	.22/.34/.25***	1		
Dbt	.72/.78/.70***	.53/.55/.40***	.18**/.26***/.17**	.38/.48/.36***	.45/.45/.36***	1	
Ult	.64/.69/.81***	.30/.55/.56***	.05/.14*/.40***	.40/.45/.39***	.22/.33/.53***	.42/.50/.53***	1

*Div* Divine, *Dem* Demonic, *Int* Interpersonal, *Mor* Moral, *Dbt* Doubt, *Ult* Ultimate meaning. ***p-values < .001; **p-values < .01

## Nomological Validity

Table 8 shows the validity-related correlations between the RSS-14 overall and its dimensions, with satisfaction, self-esteem, general health overall, and its subscales. Contrary to our hypothesis, satisfaction was inversely and significantly associated with the RSS-14 overall, doubt struggles, and ultimate meaning. The results also revealed a negative correlational tendency between life satisfaction, interpersonal, and moral strains. The lack of any association was noticed in the case of divine and demonic strains. Therefore, the hypothesis was partially confirmed. A similar pattern of the results was obtained in the case of self-esteem, which correlated negatively with the RSS-14 overall, moral, doubt, and ultimate meaning. Demonic and interpersonal strains showed an association with self-esteem at the level of tendency. The only dimension that did not correlate with self-esteem was the subscale of divine struggle. Thus, the hypothesis was not entirely confirmed.

**Table 8:** Correlations between the overall RSS-14, its subscales, life satisfaction, self-efficacy, general health, and its dimensions (Study 1–N = 250)

	RSS-14	Div	Dem	Int	Mor	Dbt	Ult
Sat	− .21**	− 0.03	− 0.03	− .10^t^	− .10^t^	− .16**	− .33***
Ses	− .28***	− 0.08	− .11^t^	− .11^t^	− .19**	− .23***	− .37***
Som	.33***	.16**	.13*	.24***	.19**	.20**	.26***
Anx	.38***	.18**	0.08	.27***	.22**	.25***	.39***
Sdy	.21**	.11^t^	0.04	0.07	0.09	.23***	.29***
Sde	.33***	.15*	0.07	.21**	.11^t^	.22***	.49***
Gho	.41***	.19**	.11^t^	.26***	.21**	.26***	.46***

*Div* Divine, *Dem* Demonic, *Int* Interpersonal, *Mor* Moral, *Dbt* Doubt, *Ult* Ultimate meaning, *Sat* Life satisfaction, *Ses* Self-esteem, *Som* Somatic symptoms, *Anx* Anxiety and insomnia, *Sdy* Social dysfunction, *Sde* Severe depression, *Gho* General health overall, ***p-values < .001; **p-values < .01; *p-*values* < .05; ^t^*p*-*values* < .1

Correlations between problematic health dimensions and the RSS-14 were positive, thus supporting the hypothesized direction. Significant and positive correlations were also found between the RSS-14, doubt, ultimate meaning and somatic symptoms, anxiety and insomnia, social dysfunction, severe depression, and general health overall. Demonic struggle correlated significantly only with somatic symptoms. Divine, interpersonal, and moral strains were associated significantly with somatic symptoms, anxiety and insomnia, and general health overall. They were not associated significantly with social dysfunction. The hypothesis was also partially confirmed.

In Table 9, validity-related associations between the RSS-14 overall and its subscales, with presence and search for meaning, stress, depression, anxiety, social desirability, God’s (dis)engagement, intellect, ideology, private practice, religious experience, public practice, and overall CRS-15 are presented. R/S struggles correlated negatively with presence of meaning and social desirability. Interpersonal struggles and ultimate meaning were negatively associated with dimensions of centrality. Positive correlations were also found between some R/S struggles and search for meaning, stress, depression, anxiety, God’s disengagement, and subscales of the CRS-15. Divine strains, doubt, and the overall RSS-14 did not correlate with the CRS-15 and its dimensions.

**Table 9:** Correlations between the overall RSS-14, its subscales, presence of meaning, search for meaning, stress, depression, anxiety, social desirability, God’s engagement, God’s disengagement, overall CRS-15, and its dimensions (Study 2–*N* = 261)

	RSS-14	Div	Dem	Int	Mor	Dbt	Ult
Mpr	− .20**	− 0.07	0.08	− .19**	0.06	− .19**	− .45***
Mse	.24***	.17**	.10^t^	.15*	.30***	.23***	0.06
Pss	.40***	.23***	.18**	.25***	.28***	.33***	.42***
Dep	.44***	.30***	.21***	.27***	.29***	.34***	.44***
Anx	.43***	.27***	.15*	.28***	.27***	.36***	.43***
Sde	− .26***	− .18**	− .12^t^	− .16**	− .15*	− .26***	− .19**
Gen	.10^t^	.11^t^	.39***	− .17**	.32***	0.05	− .11^t^
Gdi	.35***	.34***	0.03	.27***	.12^t^	.38***	.27***
Inl	.12*	0.02	.32***	− 0.07	.35***	0.09	− 0.03
Ide	0.09	.11^t^	.29***	− .21**	.30***	.11^t^	− 0.08
Prp	0.05	0.08	.36***	− .28***	.30***	0.03	− .12*
Exp	0.01	0.04	.42***	− .27***	.26***	− 0.03	− .19**
Pup	0.02	0.05	.36***	− .28***	.32***	0.02	− .19**
CRS-15	0.06	0.06	.40***	− .26***	.35***	0.04	− .15*

*Div* Divine, *Dem* Demonic, *Int* Interpersonal, *Mor* Moral, *Dbt* Doubt, *Ult* Ultimate meaning, *Mpr* Presence of meaning, *Mse* Search for meaning, *Pss* Stress, *Dep* Depression, *Anx* Anxiety, *Sde* Social desirability, *Gen* God’s engagement, *Gdi*  God’s disengagement, *Inl* Intellect, *Ide* Ideology, *Prp* Private practice, *Exp* Religious experience, *Pup* Public practice, *CRS-15* overall Religious Centrality ***p-values < .001; **p-values < .01; *p-values < 0.05; ^t^*p*-*values* < .1

From the outcomes presented in Table 10, it emerges that only two dimensions of the RSS-14—divine struggles and doubt—significantly and negatively correlate with self-efficacy. In turn, the overall RSS-14, divine struggles, moral strains, doubt, and ultimate meaning correlate positively with the overall YSQ and all its subscales. Interpersonal struggles correlate in a similar way to the variables of the RSS-14 listed above, except for two YSQ subscales—emotional deprivation and entitlement. The weakest relationships were found for demonic struggles.

**Table 10:** Correlations between the overall RSS-14, its subscales, self-efficacy, overall YSQ, and its dimensions (Study 3–*N* = 300)

	RSS-14	Div	Dem	Int	Mor	Dbt	Ult
Sef	− 0.10^t^	− .11*	− 0.08	0.01	− 0.01	− .15*	− 0.09
Emd	.28***	.23***	.17**	.11^t^	0.08	.40***	.16**
Aba	.42***	.36***	.11^t^	.18**	.26***	.50***	.31***
Mis	.38***	.31***	.12*	.17**	.16**	.50***	.27***
Soi	.37***	.23***	.11^t^	.17**	.18**	.55***	.27***
Def	.39***	.36***	.14*	.17**	.13*	.54***	.27***
Fai	.43***	.30***	0.08	.15**	.20***	.55***	.34***
Inc	.42***	.36***	.17**	.24***	.26***	.46***	.32***
Vul	.39***	.32***	.10^t^	.18**	.19**	.49***	.27***
Enm	.37***	.29***	.19**	.22***	.21***	.35***	.29***
Sub	.50***	.38***	.21***	.20***	.28***	.58***	.41***
Ssa	.37***	.22***	.14*	.20***	.28***	.39***	.28***
Emi	.35***	.26***	0.09	.18**	.15*	.46***	.29***
Unr	.36***	.23***	0.08	.20***	.31***	.39***	.27***
Ent	.24***	.17**	.10^t^	.11^t^	.13*	.31***	.17**
Ins	.31***	.16**	0.05	.17**	.23***	.44***	.20**
Adm	.35***	.24***	0.06	.20***	.22***	.41***	.28***
Pes	.43***	.31***	0.07	.23***	.22***	.58***	.30***
Spu	.45***	.38***	.19**	.19**	.28***	.49***	.32***
YSQ	.49***	.37***	.15**	.24***	.27***	.61***	.36***

*Div* Divine, *Dem* Demonic, *Int* Interpersonal, *Mor* Moral, *Dbt* Doubt, *Ult* Ultimate meaning, *Sef* Self-efficacy, *Emd* Emotional deprivation, *Aba* Abandonment, *Mis* Mistrust/abuse, *Soi* Social isolation, *Def* Defectiveness/shame, *Fai* Failure, *Inc* Incompetence/dependence, *Vul* Vulnerability to harm, *Enm* Enmeshment, *Sub* Subjugation, *Ssa* Self-sacrifice, *Emi* Emotional inhibition, *Unr* Unrelenting standards, *Ent* Entitlement, *Ins* Insufficient self-control, *Adm* Admiration seeking, *Pes* Pessimism, *Spu* Self-punitiveness, *YSQ* Overall score, ***p-values < .001; **p-values < .01; *p-values < 0.05; ^t^*p*-values < .1

## Discussion

The experience of clinical work in the last few years has mostly shown the need for a brief form of the RSS (Zarzycka et al., 2018). The aim of these studies was to conduct a Polish validation of the RSS-14 (Exline et al., 2022a), establish its internal structure, and analyze its correlations with religiousness and different dimensions of psychological and mental health factors.

With respect to the internal structure of the RSS-14, the CFA from three separate studies confirmed the good fit of the six-factor model. The goodness-of-fit indices were very similar to those obtained in the original version of the tool. Both the total score and the subscales had high reliability and acceptable stability over the three studies.

With respect to the nomological analyses, we assumed a lack of connection between the RSS and life satisfaction (Study 1). Our predictions were partially confirmed as it was observed that only two categories of religious struggles (doubt and ultimate meaning) and the overall RSS was related negatively to life satisfaction. Given the low average age of our study group and the low intensity of religious struggles in young people (Ellison & Lee, 2010), we can conclude that worrying because of doubts and questions about one’s own R/S beliefs, and religious concern caused by the loss of deep meaning and sense of life was associated with lower life satisfaction. Another study showed that in reference to a specific struggle, negative attributions of God’s intent, less meaning found, and greater perceived spiritual decline predicted a poorer profile of well-being (Wilt et al., 2017).

The hypothesis related to the relationship between meaning in life and spiritual struggle was also partially confirmed. Our study revealed a different character of the association between R/S struggles and presence and search for meaning in life (Study 1). This regularity was noted not only for the global effect of the religious struggle but also for moral and doubt struggles. This is the same result the researchers obtained when constructing the 14-item form of the Religious and Spiritual Struggles Scale (Exline et al., 2022a). Such an outcome provides evidence that the Polish adaptation of the RSS-14 is a valuable tool for measuring religious tensions and that it can be used in research on functional aspects of religiosity. Such categories of struggle as divine, interpersonal, moral, doubt, and global struggle were positively associated with the search for meaning. This can mean that people experiencing difficulties related to faith are less able to make sense of what they are facing in a way that is satisfactory to them (searching for meaning). Surprisingly, at the same time, the tension associated with religiosity can also serve the function of making meaning (presence meaning). In the case of people who have an established meaning in life, religious struggles become a contribution to finding an answer to the question related to the meaning of life (Szcześniak et al., 2022b).

Our research on the relationship of R/S struggles with health symptoms (Study 1) has shown that all categories of religious difficulties have positive associations with poorer health indicators and sleep problems. Identical results were obtained by the authors of the brief version of the RSS-14 (Exline et al., 2022a). This result is both intuitive and consistent with many research findings in this respect. R/S struggles are often linked with negative mental health outcomes, including poor emotional adjustment to stress, depression, fear, anxiety, and guilt. The strongest relationships were obtained between the symptoms of depression and this category of religious difficulties, which concerns the obstacles experienced by the person in the tension around ultimate meaning.

In Study 1, all types of religious struggles correlated positively with stress. Such clear and strong associations between stress and all measures of religious struggles make us understand this measure of religiosity in highly stressful terms. Our research confirms a well-established regularity in psychology that feeling “spiritually empty” and distant from God is associated with higher levels of emotional exhaustion, which is also caused by the experience of stress. Such relationships are indicated by the RSS-14 in both the US population (Exline et al., 2022a) and the Polish sample.

Contrary to our assumption, and in opposition to our earlier studies (Szcześniak et al., 2022a), no links were found between self-esteem and all measures of religious struggles (Study 2). This may be because people experiencing difficulties in faith focus more on solving spiritual and religious dilemmas than on themselves, their potential, and resources. They do so by engaging more in their own experience and relationships with the Sacred than on the isolated focus of their predispositions.

The association between the RSS-14 and other measures of religiosity showed some interesting results. Study 2 did not reveal relationships between the divine, doubt, and CRS-15. Most likely, people do not admit and do not want to disclose the negative emotions and distrust they hold toward God. Maybe the unpleasant emotions they experience are not interpreted in terms of religious tensions. Previous research pointed to this problem. People are reluctant to acknowledge their negative emotions toward God because they see them as bad in terms of morality.

We noticed similar relationships in the case of social desirability (Study 2) as the authors of the US RSS-14 method. People do not treat R/S struggles as a form of social self-presentation, and they are more ashamed of them (Exline et al., 2022a). On the one hand, believers can cope with the existence of religious doubts, and even when they arise, it does not involve their cognitive-emotional system excessively. On the other hand, faith involves entering crisis, which is fully natural. Moreover, there were strong relationships between demonic struggle and religiosity. This may suggest that R/S struggles are present in religiosity even if people don’t admit them. The beliefs of the major religious systems assume the existence of evil, which takes on more defined diabolical forms or ones less specified, but still some kind of supernatural evil (Wilt et al., 2017). Ultimate meaning has a negative relationship with the CRS-15. This result, firmly established in the literature on the meaning-making function of religiosity (Park et al., 2013), was confirmed in the present study. Higher levels of involvement in religiosity predicted higher moral struggles (Exline et al., 2022a).

It turned out that perceiving God as not listening to people’s prayers connects to most types of R/S struggles (Study 2). God’s disengagement was related to the notion of: 1) a God who disconnects from a person; 2) people who are committed to the faith and despite that hurt others; 3) doubts about R/S beliefs, and 4) concern that centers on a lack of perceived deep meaning in life. We also obtained similar results to those of the original version of the RSS-14 in this respect. Believing in a God who is attentive to prayers is also associated positively with two types of religious struggle—demonic and moral*.* It may be easier for people to fight a spiritual battle against evil and struggle with the moral aspects of religiosity if one is aware of God’s presence in prayer (Szcześniak et al., 2021)*.*

The exploration of R/S struggles with the category of cognitive schemas was the new element of our research (Study 3). As we assumed, religious struggles were positively linked to cognitive patterns which were understood as a mental representation containing organized, prior knowledge about a particular domain, including a specification of the relations among its attributes (Bjorck, 1995). This may suggest that religious beliefs can provide a cognitive map of the world, which can make it meaningful (Barrett & Zahl, 2013). Our findings can be taken as a suggestion to look at R/S struggles from a slightly different perspective as before psychology did. In this case, it is possible to reflect on the subject of R/S struggles as a construct, which is not just a religious form of thinking activated in the more difficult moments of a person’s life, but a more profound schema that has been built up through interactions with the environment, and modified by experience and a not always easy relationship with the divine (Bjorck, 1995). It can be concluded that R/S struggles are involved in people’s interpretation of one’s life. This means that it is much more legitimate to use a shortened version of the RSS to study religiosity and its relationship to negative aspects of human functioning. These analyses showed good support for the nomological validity of the proposed subscales.

## Limitations

Limitations in this study are related to the correlational model used and the cross-sectional nature of the research. Future directions involve the need to explore the dynamics of changes in religious struggles based on measurement with RSS-14. In addition, although the study as a whole included three relatively large samples (811 respondents in total) from the Polish population, Catholics predominated (according to the social structure), agnostics and atheists were also included. Little presence of Orthodox people, Protestants or Polish Muslims (in the category of religious affiliation “Other”, Table 1) raises further questions about whether the structure of religious struggles is also replicable in groups recruited from other Christian denominations as well as religions. The limitation related to validation is the transfer of a ready-made poll of items, created in a different culture and language. Further research on measuring religious struggles should move toward building a new, culturally tailored tool.

## Conclusions

In our three-part study, we have translated and validated a short measure for religious struggles, the RSS-14. Based on the results of our research, the Polish version can be assessed as a valid and reliable tool. In addition, we extended these analyses with nomological validation, which indicated negative associations of religious struggles with life satisfaction, presence of meaning, social approval, self-esteem, the centrality of religiosity, and positive with God’s disengagement, search for meaning in life, weaker mental health indices, sleep problems, and stress. The relationships discovered are congruent with those obtained by the authors of the original version of the scale. Moreover—which is a novelty of our project—we have found negative correlations with early cognitive schemas. This outcome stands for a significant indication to use the scale for scientific research on deepening the religious aspects of schema therapy. All of this convinces us that the validated tool can help in the study of the phenomenon of religious struggles in the Polish context.

## Data Availability

All data and materials as well as software application or custom code support their published claims and comply with field standards.

## Materials and Code Availability

The datasets used during the current study are available from the corresponding author.
